# Phylogeography and Post-Glacial Recolonization in Wolverines (*Gulo gulo*) from across Their Circumpolar Distribution

**DOI:** 10.1371/journal.pone.0083837

**Published:** 2013-12-30

**Authors:** Joanna Zigouris, James A. Schaefer, Clément Fortin, Christopher J. Kyle

**Affiliations:** 1 Environmental and Life Sciences Gradate Program, Trent University, Peterborough, Ontario, Canada; 2 Biology Department, Trent University, Peterborough, Ontario, Canada; 3 Carcajou Québec, Stoneham-et-Tewkesbury, Québec, Canada; 4 Forensic Science Department, Trent University, Peterborough, Ontario, Canada; Institute of Biochemistry and Biology, Germany

## Abstract

Interglacial-glacial cycles of the Quaternary are widely recognized in shaping phylogeographic structure. Patterns from cold adapted species can be especially informative - in particular, uncovering additional glacial refugia, identifying likely recolonization patterns, and increasing our understanding of species’ responses to climate change. We investigated phylogenetic structure of the wolverine, a wide-ranging cold adapted carnivore, using a 318 bp of the mitochondrial DNA control region for 983 wolverines (*n* = 209 this study, *n* = 774 from GenBank) from across their full Holarctic distribution. Bayesian phylogenetic tree reconstruction and the distribution of observed pairwise haplotype differences (mismatch distribution) provided evidence of a single rapid population expansion across the wolverine’s Holarctic range. Even though molecular evidence corroborated a single refugium, significant subdivisions of population genetic structure (0.01< Φ_ST_ <0.99, *P*<0.05) were detected. Pairwise Φ_ST_ estimates separated Scandinavia from Russia and Mongolia, and identified five main divisions within North America - the Central Arctic, a western region, an eastern region consisting of Ontario and Quebec/Labrador, Manitoba, and California. These data are in contrast to the nearly panmictic structure observed in northwestern North America using nuclear microsatellites, but largely support the nuclear DNA separation of contemporary Manitoba and Ontario wolverines from northern populations. Historic samples (c. 1900) from the functionally extirpated eastern population of Quebec/Labrador displayed genetic similarities to contemporary Ontario wolverines. To understand these divergence patterns, four hypotheses were tested using Approximate Bayesian Computation (ABC). The most supported hypothesis was a single Beringia incursion during the last glacial maximum that established the northwestern population, followed by a west-to-east colonization during the Holocene. This pattern is suggestive of colonization occurring in accordance with glacial retreat, and supports expansion from a single refugium. These data are significant relative to current discussions on the conservation status of this species across its range.

## Introduction

Understanding phylogeographic differentiation and patterns of genetic diversity for many extant species in the Northern Hemisphere hinges on understanding the interglacial-glacial cycles of the Quaternary [1], [2]. Cold adapted species can be particularly informative as these species typically experience population increases and range expansions during glacials, and range contractions during interglacials [1], [3]. These species may also undergo range contractions into refugia when continental ice-sheets attain their greatest extent (e.g. [4]), further shaping present-day patterns of biodiversity in the arctic [5].

Refugial history has focused predominately on temperate taxa [6], revealing major refugia – like Beringia [7]. However, progressively more investigations are proposing ‘cryptic’ glacial refugia beyond the limit of glaciations (see review by Shafer et al. [2]). Such refugia are usually limited and sporadic in geographic extent, and often overlooked in glacial biogeographic reconstructions based on fossil records [8], [9]. However, identification of the same regions by multiple studies (see Beatty and Provan [10]) signifies these refugia are not so much cryptic as additional to conventional Pleistocene refugia [11]. For many taxa, isolation of populations in separate refugia resulted in the formation of distinct genetic lineages [6], [12]. Patterns of genetic differentiation are also reflective of stochastic processes like genetic drift and species specific dispersal abilities [13]. Wide-ranging species generally display a lack of population structuring across their range, increasing the likelihood of similar mitochondrial haplotypes being observed across very distant geographical locations [14], [15]. Overall, these data are relevant to understanding how historic processes influence contemporary genetic patterns and how these data should be interpreted in context of management actions for species of conservation concern.

Most phylogeographic studies of cold adapted taxa have focused on vegetation [16], birds [17], and small mammals [18]. Investigations of large mammals remain limited [19], [20], even though large body size (>5.5 kg) is a strong predictor of extinction risk for mammals [21]. Furthermore, relic populations may contain biogeographic traits associated with glacial refugia [22], particularly at range peripheries. This has conservation implications where peripheral populations – faced with increased environmental fluctuations [23], reduced gene flow and low densities [24] – may maintain unique genetic variability necessary in responding to climate change [25].

**Figure 1:**
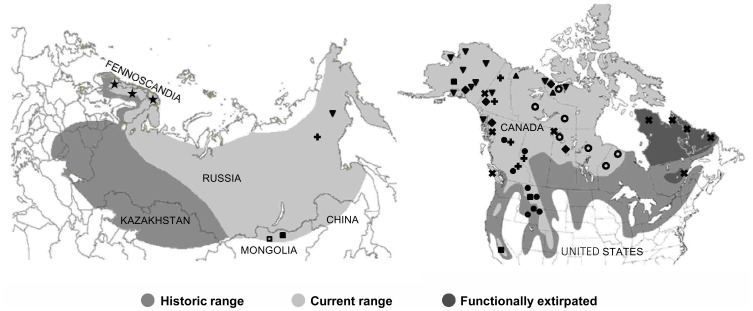
Historic and contemporary Holarctic distribution of wolverines and sampling localities. Historic and current distribution of wolverines in Eurasia (adapted from National Geographic Society [116]) and North America (adapted from COSEWIC [115]), and sampled localities from Wilson et al. [45] - *triangle*; Walker et al. [15] - *star*; Tomasik and Cook [55] - *inverted triangle*; Cegelski et al. [49] - *solid circle*; Schwartz et al. [60] - *solid square*; Frances [59] - *diamond*; Zigouris et al. [51] - *open circle*; Rochnov and Meschersky unpub. - *open square*; New contemporary samples - *plus sign*; and New historic samples - *x symbol*.

Wolverines (*Gulo gulo*) are a cold adapted carnivore with a circumboreal distribution (Figure 1, [26]). Fossil evidence is minimal for this species [27], and from those records that do exist, fossils in Europe extend from the Iberian Peninsula [28] eastwards to the Czech Republic [29]. In North America, late Pleistocene fossil remains have been found in Alaska [30] and the Yukon Territory [31]. Given the gaps in the fossil record, genetic evidence could provide a clearer picture of how glacial refugia have shaped postglacial recolonization of wolverines and other cold adapted species.

In the past half-century, wolverines have experienced substantial range reductions along the southern edge of their circumpolar distribution due to habitat alterations, persecution, and other anthropogenic influences such as indirect poisoning campaigns targeted at wolves (Figure 1, [26], [32], [33]. In North America, this animal has lost 37% of its historic range [34]. Although globally a species of least concern [35], its regional status ranges from stable (Alaska, [36]) to endangered (Norway, [37]), and in some areas, functionally extirpated (Quebec-Labrador, [38]). Wolverines occur at low densities [39] and have very large home range sizes (188–2 563 km^2^ for males, [40], [41]), increasing their vulnerability to habitat fragmentation and external anthropogenic threats such as logging, fur harvesting and direct persecution [42]–[44].

Wolverines are highly vagile [40], [41], largely explaining the high levels of gene flow observed among populations based on nuclear DNA [45]–[49]. Genetic structure, however, tends to increase towards range peripheries in North America [46], [47], [49]–[51], suggesting irregular distributions of populations [36] due to range contractions [44]. Additionally, spring snow cover is positively correlated with wolverine distribution [52], [53] and genetic differentiation [54], underlining the sensitivity of wolverines to climate change. Mitochondrial DNA (mtDNA) studies of this species have revealed strong genetic structure over small geographic scales, reflecting female philopatry (e.g. [49], [55]). In North America, haplotypes from the eastern periphery drastically differed from those in the west, suggesting a longstanding subdivision reflective of historical processes [51].

Here, we investigated the phylogeographic patterns of wolverines across the full breadth of their Holarctic range (>5 million km^2^) using conventional Bayesian approaches. We obtained samples from 209 individuals together with existing data from 774 wolverines to assess the genetic variation of the mtDNA control region. In addition, we apply a coalescent-based approximate Bayesian computation (ABC) method to test among competing hypotheses of postulated recolonization pathways influencing population divergence. We propose four alternative hypotheses that may explain observed patterns of haplotype distribution and resulting groupings that include: (1) present-day populations diverged from a single ancestral population during the last glacial maximum (LGM); (2) a single incursion across Beringia resulted in the divergence between Eastern and Western Hemisphere wolverines, with divergence of North American populations occurring during glacial retreat; (3) a single incursion across Beringia during the LGM, followed by a west-to-east stepping-stone divergence pattern across North America; and (4) two incursions from Beringia during the LGM, with the second incursion being followed by a west-to-east stepping-stone divergence. Understanding the response of cold adapted species to past climatic fluctuations could help identify evolutionary significant units [56] and improve predictions of the effects of climate change on arctic wildlife [57].

## Methods

### Samples

We processed contemporary samples collected from: Russia (RUS, *n* = 49), Yukon (YK, *n* = 26), British Columbia (BC, *n* = 81), Alberta (AB, *n* = 26) and Manitoba (MB, *n* = 1), and historical samples (Figure 1, Table S1) collected between 1889–1944 as identified by the Global Diversity Information Facility (http://www.gbif.org/) from the Yukon (*n* = 9, c. 1923–1932), Nunavut (NU, *n* = 1, c. 1944), British Columbia (*n* = 11, c. 1910–1927; with one sample representing *G. gulo vancouverensis* from Vancouver Island, Canada), Saskatchewan (SK, *n* = 1, c. 1920), Ontario (ON, *n* = 1, c. 1920) and Quebec-Labrador (QC/NL, *n* = 15, c. 1883–1900). Contemporary samples for Russia, Yukon, Alberta, and Manitoba were collected from pelts through fur auction houses and pelt dealers. British Columbia samples were obtained from pelt samples through fur auction houses, and tissue samples (ear plug and hair) collected by the Columbia Basin Fish and Wildlife Compensation Program. Permission to acquire tissue samples from archived specimens was obtained from all museums and institutions, and all tissue samples from historic specimens were donated. Our data were combined with mtDNA control region data from previous studies (Figure 1, [15], [45], [49], [51], [55], [58], [59], Rochnov and Meschersky unpublished data) – including samples from: Sweden (SWE, *n* = 62), Norway (NOR, *n* = 108) Mongolia (MNG, *n* = 6), Russia (*n* = 5), Alaska (AK, *n* = 148), Yukon (*n* = 23), Nunavut (*n* = 81), Northwest Territories (NT, *n* = 53), British Columbia (*n* = 5), Saskatchewan (*n* = 16), Manitoba (*n* = 30), Ontario (*n* = 54), Montana (MT, *n = *148), Wyoming (WY, *n* = 13), Idaho (ID, *n* = 15), and California (CA, *n* = 7). We did not include data reported in Cegelski et al. [49] for BC and AB, and for ON reported by Frances [59] as samples were obtained from C.J. Kyle and likely the same individuals. We also excluded sequence data from Chappell et al. [48] as the mtDNA control region was 200 bp and did not encompass five known variable sites in the larger fragment.

### Preparation of Bone Samples and DNA Extraction

Bone dust (∼100 µL) was collected from museum specimens by drilling mandibles or from turbinate bones frozen with liquid nitrogen and crushed into bone powder using a mortar and pestle. Mandibles were initially washed with Decon solution (1∶49), rinsed with DNAase-free ddH_2_O (Gibco), and the outer surface removed using a Dremel tool. Strict laboratory protocols were followed to minimize risk of cross-contamination from contemporary sources. Specifically, equipment was sterilized with Decon solution (1∶9) and rinsed with DNAse-free water between handling each sample, and filter tips and disposable pipettes were used. Extraction blanks were included at the beginning and end of extractions, and after every fifth sample to assess sample cross-contamination. Historic samples were processed in rooms not exposed to contemporary samples.

Extraction procedures followed the manufacturer’s protocol for Qiagen’s DNeasy tissue extraction kit (Qiagen) for all contemporary samples, with the following modifications for historic samples: (1) historic samples were rotated overnight at room temperature in 1.5 mL of ethylenediaminetetraacetic acid (EDTA; 0.5 M, pH 7.5) to decalcify the bone powder, and after 24 h samples were centrifuged at 9 000 rpm for 60 s, EDTA was poured off and a second treatment applied; (2) used 1.5 mL of Buffer ATL and treated samples with a second dose of 20 µL Proteinase K (600 mAU/mL) (Qiagen, Mississauga), followed by a 2 h incubation at 56°C; (3) incubated for 10 min at 56°C after adding buffer AL; (4) performed two elution steps with heated buffer at 70°C; and (5) transferred both elutions to an Amicon Ultra-0.5 centrifugal unit that yielded approximately 20 µL of DNA.

### Mitochondrial DNA Sequencing

A 360 bp fragment of the control region was independently amplified twice using primers Gulo0F [60] and H16498 [61]. Amplification of a shorter sequence length allowed for data from previous studies to be compared with our data set. Sequencing procedures followed Zigouris et al. [51], however, for the historic samples Taq was increased from 0.05 to 0.1 U/µL and PCR cycles increased from 30 to 50. Additionally, PCR products that showed extra banding were run on a gel, and the target band was cut out. Excised bands were frozen overnight at −80°C, vortexed and centrifuged. Amplified DNA expelled from excised gel bands and from samples with low DNA concentrations were re-amplified a second time to confirm sequence data.

### Data Analyses

Sequences were edited with MEGA 4.0.2 [62], aligned with Clustal W [63], and verified visually. We identified variable nucleotide positions and compiled unique sequences using FABOX 1.35 [64]. New haplotypes were only confirmed when independent PCR reactions generated the same sequence. Historic and contemporary samples were both treated as separate entries (historic SK omitted due to *n* = 1), as well as combined by sampling region. In this study, historic and contemporary samples are reflective of sampling occurring pre- and post-20th century population declines [65]. Thus, frequency differences between pre- and post-population declines may provide insight of the underlining processes influencing present-day patterns of haplotype distribution and frequency. Nucleotide (π) and haplotype (*h*) diversity values were estimated with ARLEQUIN 3.5 [66]. Haplotype richness and number of private haplotypes were calculated manually and with a rarefaction test (ADZE 1.0, [67]) based on the smallest sample size. We tested for departures from neutrality (Tajima’s *D*, [68]) and population growth (Fu’s *Fs*, *P<*0.02, [69]) using ARLEQUIN with 10 000 bootstrap replicates.

Differentiation among sampling sites were estimated with pairwise Φ_ST_ values using ARLEQUIN (10 000 permutations, *P*<0.05). An analysis of molecular variance (AMOVA) was used to quantify genetic variability among groups of populations, as well as within and among populations [70], in ARLEQUIN for 10 100 permutations. Groupings reflected the sharing of haplotypes among sampled regions irrespective of geographic location. We evaluated optimal grouping of sites by defining groups of samples maximally differentiated, but geographically proximate to each other. We performed a spatial analysis of molecular variance (SAMOVA) with SAMOVA 1.0 [71], 2≤ *K* ≤10, using 100 random initial conditions and 10 000 iterations. Historic samples from YK, BC and SK were omitted from SAMOVA because they represented some of the main haplotypes observed in contemporary samples. SAMOVA analyses were performed with and without historic samples from CA and QC/NL for all contemporary data and for North American samples only. Historic samples from CA and QC/NL were included, as contemporary data was absent from these two peripheral regions. We also conducted Mantel tests [72] using Isolation by Distance (IBD) Web Service 3.23 [73] to test for correlations between genetic and geographic distances. To minimize error in characterizing IBD [74], we excluded sample regions with *n* <10 individuals. Correlations were tested between the natural logarithm of geographical distances and the regression of paired Φ_ST/_(1 - Φ_ST_) estimates as proposed by Rousset [75]. Mantel tests were performed on North American regions and the data as a whole. For Eurasia, there were too few distance classes for IBD to be detected [76].

We performed Bayesian analyses in BEAST 1.7.1 [77] to investigate phylogenetic composition of lineages and estimate divergence times (e.g. [78]). The Hasegawa-Kishino-Yano (HKY, [79]) model with a gamma shape distribution and invariant sites best fit our data based on Akaike Information Criterion (AIC) values (jModelTest 0.1.1, [80]). Base frequencies were estimated and rate variation among sites was modeled using four gamma rate categories. Divergence estimates were calculated using two independent calibration points [81], averaged across two phylogenetic studies ([82], [83]: divergence between the *Gulo/Martes* clade and *Mustela* 11.9 (9.9–14.1, 95% CI) million years ago (Mya); and divergence of *Gulo* and *Martes* 6.1 (4.3–8.1, 95% CI) Mya). Control region sequences of mtDNA for *Mustela putorius*, *M. frenata* and *M. nivalis* represented the Mustelidae outgroup (GenBank Accession Numbers: AY962032; HM106321; HM106319), and *Martes martes* was used for the *Martes* outgroup (Accession Number: AJ585357). We used an uncorrelated lognormal relaxed clock model with a Coalescent constant size tree and normal distribution priors. Analyses were run for 40 million generations, with a burn-in of the first 4 million, and a sampling frequency of 500 steps. Tracer 1.5 [84] was used to evaluate estimated values and effective sample size (ESS) for each model parameter. For all parameters, ESS >450 suggested sufficient sampling and acceptable mixing. Bayesian analyses were computed multiple times to check for convergence. The phylogenetic tree was constructed using TreeAnnotator with a 20% burn-in of total trees generated, and viewed in FigTree 1.3.1 [85]. A median-joining network was created to visualize haplotype relationships using Network 4.5 [86].

To test for population expansion, we used ARLEQUIN with 10 000 bootstrap replicates to estimate the distribution of observed pairwise haplotype differences (mismatch distribution, [87]) among individuals. Populations that have undergone recent expansion display a unimodal distribution, whereas populations at demographic equilibrium are multimodal [87]. The validity of the estimated expansion model was evaluated using the sum of squared deviations (SSD) between observed and expected mismatch distribution values [88], and the raggedness index (*r,* [89]) that measures the smoothness of the mismatch distribution. Given the low power of *r* to detect population expansions, we also calculated Fu’s *Fs* statistic on the whole dataset.

**Figure 2:**
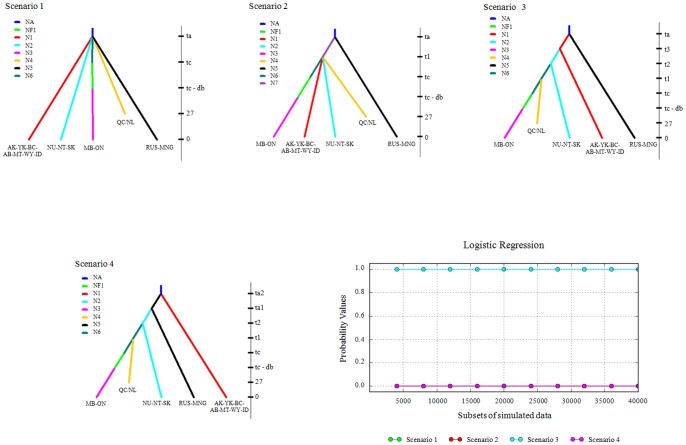
Scenarios used in DIYABC and logistic regression. Graphic representation of the four scenarios used in DIYABC and logistic regression of posterior probabilities with a recent bottleneck (20th century) for MB-ON. Please note that time is not to scale.

Based on the results of the preceding analyses, we assumed that all populations had the same colonization origin, and likely located near Beringia. We compared four alternative colonization pathways (Figure 2) using the coalescent-based approximate Bayesian computation (ABC, [90]) in DIYABC v2.0 (Cornuet et al. [91]). The first two hypotheses take into account the wolverine’s vagility and adaptation to cold environments, and postulate that colonization included regions that were not entirely ice-free. Under this assumption, individuals from the ancestral population could have dispersed in multiple directions, colonizing several regions simultaneously (e.g. [92]). The first scenario tests if all populations diverged from a single ancestral Holarctic population during the LGM, where the incursion across Beringia and multiple colonization events occurred concurrently. The second scenario includes a time lag between the single incursion event across Beringia during the LGM, and multiple colonization events. The third and fourth hypotheses assume that colonization was constrained by glaciers, and dispersing individuals followed retreating ice-sheet fronts. The third hypothesis tests a single incursion from Beringia during the LGM giving rise to a northwestern AK-YK-BC-AB-MT-WY-ID population (Pop1), from which colonization occurs in a west-to-east stepping-stone direction during the Holocene. This would result in a ‘leading edge’ pattern of colonization, with decreasing genetic diversity further away from the putative refugium [93]. Given the likelihood of multiple incursions across Beringia (e.g. red fox, *Vulpes vulpes*, [94]), our fourth hypothesis tests the occurrence of two incursions across Beringia during the LGM, resulting in the divergence between RUS-MNG (Pop5) and AK-YK-BC-AB-MT-WY-ID (Pop1) and between Pop 5 and NU-NT-SK (Pop2). This second incursion was then followed by a sequential, eastward stepping-stone dispersal scenario during the Holocene. We conducted ABC analyses for all regions except for SWE, NOR, and CA_H_. These localities were excluded based on either a large geographic gap in information between sampled regions (e.g. SWE-NOR and RUS-MNG) or small sample size (e.g. CA_H_, *n* <10). Although wolverine populations in North America experienced declines during the 20th century due to predator control programs and fur trading [65], we assumed that only Pop3 (MB-ON) experienced a genetic bottleneck given the low abundance of wolverines in this region. We applied a 75% reduction in the effective population size (*Ne*), thus simulating an extreme bottleneck [95]. Simulations for all scenarios were performed with and without this bottleneck. For all scenarios, the sex ratio was equal and generation time was four years [36]. Range limits and associated conditions of parameters used in DIYABC analyses are listed in Table S2. We simulated 10^6^ data sets for each scenario to build a reference table. The closest 1% of the simulated datasets to the observed data was used to estimate relative posterior probabilities per scenario. Type I and type II errors were calculated from 500 simulated data sets per scenario using the same prior distribution parameter values as the scenarios. Because Bayesian posterior probabilities were used, generated type I and type II errors are not reflective of a classical frequestist hypothesis framework, where the null is never accepted but rejected when data are incompatible with it [96]. When type II errors are small under the ABC approach there is good confidence in the results, even though type I errors can be large [97].

## Results

### Haplotype Diversity

We sequenced 360 bp of the control region for 183 contemporary samples and 26 out of 38 extracts for museum specimens (Table S1). Analyses were performed on 318 bp to include data from Walker et al. [15]. Three variable sites were lost when the 360 bp fragment was shortened to 318 bp. However, the inclusion of Walker et al. [15] data added two different variable sites, resulting in the overall loss of a single variable site. Haplotypes ‘C’ and ‘J’ from Tomasik and Cook [55] were amalgamated with haplotypes ‘A’ and ‘F’ from Wilson et al. [45], respectively (Table S3). In addition, haplotype ‘Mong1’ from Schwartz et al. [60] grouped with haplotype ‘L’ from Tomasik and Cook [55]. All three amalgamated haplotype pairs differed by one base pair. We compared all haplotypes to reports in the literature [45], [48], [49], [51], [55], [59]. Although multiple studies identified identical haplotypes, naming was inconsistent among publications. All haplotypes identified for the control region of *G. gulo* were catalogued by publication date (Table S3).

**Figure 3:**
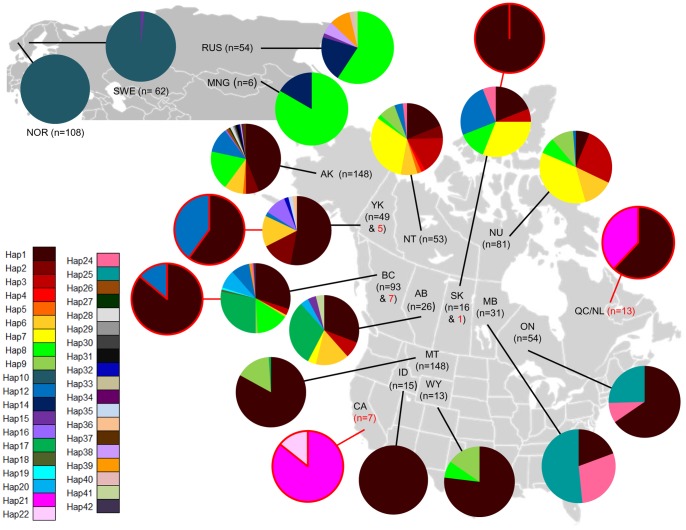
Geographical distribution of mtDNA haplotypes. The geographic distribution of mtDNA control region haplotypes based on frequencies observed for each region. Historic samples are presented separately and identified with a red border and red sample size number.

A total of 39 haplotypes and 28 variable sites were identified from 983 individuals (Table S4), with 34 haplotypes observed in previous studies. All five new haplotypes identified in this study were sequenced from pelt samples, with the majority of the samples tested having high molecular weight DNA, and confirmed with independent PCRs. Voucher sequences of new haplotypes were submitted to GenBank (Accession Numbers: KC182788 - KC182792). The most common haplotype was Hap1, found within 36% of all individuals. Hap1 was only observed in the Western Hemisphere, comprising 46% of North America samples and even occurring on Vancouver Island. The next most frequent haplotypes were Hap10 and Hap8, comprising 17% and 9% of all samples, respectively (Figure 3, Table S5). Fourteen of the remaining haplotypes occurred at frequencies between 1–5% and represented 36% of the data. The remaining 22 haplotypes were considered rare, each <1% and overall comprising 2% of the data (Figure 3, Table S5).

Population genetic parameters were consistent among groupings of contemporary and historic samples, but we present only results where contemporary and historic samples were treated as separate entries. Nucleotide diversity (π) was low across all regions, but highest in MB and ON (Table 1). Haplotype diversity (*h*) ranged from 0 (NOR and ID) to 0.83 (NT and SK), and was highest in northwestern North America and Russia (Table 1). Accounting for sample size (standardized to *g*_CONTEMPORARY_ = 6, and *g*_HISTORIC_ = 5), private haplotypes were highest in YK and RUS, and absent from NOR, WY, ID, BC_H_, and QC/NL_H_ (0.00 to 1.16, Table 1). All tests, with the exception of two, produced non-significant values for both Tajima’s *D* and Fu’s *Fs* (Table 1). Significant values were observed for Tajima’s *D* (−1.44, *P = *0.04) for Sweden and for Fu’s *Fs* (−5.46, *P = *0.04) for Alaska.

**Table 1:** MtDNA nucleotide diversity (*π*), haplotype diversity (*h*), haplotype richness (*H*_R_), private haplotype (*H*_P_) counts, their standard deviations (*SD* π, *SD h*, *H*_Rstd_, and *H*_Pstd_) and standardized to the smallest sample size for both contemporary (*g* = 6) and historic (*g* = 5) samples using ADZE rarefaction, Tajima’s *D*, and Fu’s *Fs*.

	Region	*n*	π	*SD* π	*h*	*SD h*	*H*_R_	*H*_Rstd_	*H*_P_	*H*_Pstd_	Tajima’s *D*	*P*	Fu’s *Fs*	*P*
Contemporary	SWE	62	<0.001	0.001	0.03	0.03	2	1.10	0	0.07	−1.44	0.04	−0.57	0.14
	NOR	108	0	0	0	0	1	1	0	0	0	1	–	–
	MNG	6	0.002	0.002	0.33	0.22	2	2	0	0.24	−1.13	0.15	0.95	0.61
	RUS	54	0.003	0.003	0.61	0.06	7	2.86	2	1.00	−0.08	0.52	−1.56	0.21
	AK	148	0.006	0.004	0.75	0.03	17	3.64	9	0.77	−0.59	0.31	−5.46	0.04
	YK	49	0.003	0.003	0.68	0.06	8	3.23	2	1.16	−0.74	0.27	−2.72	0.07
	NT	53	0.006	0.004	0.83	0.03	11	4.10	1	0.42	0.53	0.74	−2.97	0.09
	NU	81	0.004	0.003	0.78	0.03	7	3.63	0	0.20	0.60	0.75	−0.69	0.40
	BC	86	0.006	0.004	0.80	0.02	13	3.84	3	0.65	−0.08	0.53	−3.39	0.09
	AB	26	0.005	0.004	0.81	0.05	8	3.85	0	0.68	0.14	0.60	−2.10	0.10
	SK	16	0.007	0.005	0.83	0.06	6	3.99	0	0.19	1.46	0.93	−0.26	0.45
	MB	31	0.013	0.007	0.63	0.05	3	2.65	0	0.40	2.49	0.99	8.07	0.99
	ON	54	0.009	0.005	0.51	0.06	3	2.31	0	0.03	1.42	0.92	6.83	0.98
	MT	148	0.003	0.002	0.30	0.04	3	1.74	0	0.20	0.11	0.62	2.47	0.87
	WY	13	0.003	0.003	0.41	0.15	3	1	0	0	−0.48	0.30	0.98	0.71
	ID	15	0	0	0	0	1	1	0	0	0	1	–	–
Historic	YK	5	0.008	0.006	0.60	0.18	2	2.00	0	0.29	1.57	0.96	3.02	0.91
	BC	7	0.004	0.003	0.29	0.20	2	1.71	0	0	−1.36	0.08	2.05	0.83
	QC/NL	13	0.008	0.005	0.51	0.08	2	1.96	0	0	2.11	0.99	5.45	0.99
	CA	7	0.001	0.001	0.29	0.20	2	1.71	1	0.76	−1.01	0.23	−0.09	0.23

SWE = Sweden; NOR = Norway; MNG = Mongolia; RUS = Russia; AK = Alaska; YT = Yukon; NT = Northwest Territories; NU = Nunavut; BC = British Columbia; AB = Alberta; SK = Saskatchewan; MB = Manitoba; ON = Ontario; QC/NL = Quebec-Labrador; MT = Montana; WY = Wyoming; ID = Idaho; CA = California.

### Genetic Structure

Pairwise Φ_ST_ estimates (Table 2) revealed several differentiated groups. In Eurasia, wolverines were not genetically differentiated between SWE and NOR (Φ_ST = _0.01, *P* = 0.36), nor between MNG and RUS (Φ_ST_ = −0.06, *P* = 0.79). However, wolverines from SWE and NOR had high levels of divergence with MNG and RUS (Φ_ST_ ≥0.89), and all four were differentiated from North America (Φ_ST_ ≥0.21; Table 2). In North America, five main divisions were observed based on the pairwise Φ_ST_ estimates that largely pooled the Arctic regions (NT, NU, northern SK), western regions (AK, YK, BC, AB, MT, WY, ID), and eastern regions (ON, QC/NL_H_). The additional two divisions included MB and CA_H_ that were differentiated from the other North American regions (Table 2), with divisions being more pronounced for CA_H_.

**Table 2:** Pairwise estimates of population genetic distance for mtDNA among sampling localities (Φ_ST_, below diagonal), and associated *P* values (above diagonal).

	SWE	NOR	MNG	RUS	AK	YT_C_	YT_H_	NT	NU	BC_C_	BC_H_	AB		SK	MB	ON	QC/NL_H_	MT	WY	ID	CA_H_
SWE	•	0.36	0.00	0.00	0.00	0.00	0.00	0.00	0.00	0.00	0.00	0.00	0.00		0.00	0.00	0.00	0.00	0.00	0.00	0.00
NOR	0.01	•	0.00	0.00	0.00	0.00	0.00	0.00	0.00	0.00	0.00	0.00	0.00		0.00	0.00	0.00	0.00	0.00	0.00	0.00
MNG	0.97	0.99	•	0.79	0.00	0.00	0.00	0.00	0.00	0.00	0.00	0.00	0.01		0.03	0.01	0.03	0.00	0.00	0.00	0.00
RUS	0.89	0.93	−0.06	•	0.00	0.00	0.00	0.00	0.00	0.00	0.00	0.00	0.00		0.00	0.00	0.00	0.00	0.00	0.00	0.00
AK	0.81	0.84	0.33	0.40	•	0.00	0.33	0.00	0.00	0.00	0.40	0.01	0.00		0.00	0.00	0.00	0.00	0.30	0.00	0.00
YT_C_	0.92	0.95	0.60	0.61	0.06	•	0.02	0.00	0.00	0.00	0.40	0.00	0.00		0.00	0.00	0.00	0.00	0.21	0.03	0.00
YT_H_	0.67	0.99	0.53	0.61	0.01	0.23	•	0.06	0.00	0.09	0.53	0.05	0.25		0.06	0.14	0.05	0.04	0.29	0.06	0.00
NT	0.86	0.91	0.31	0.41	0.14	0.33	0.13	•	0.04	0.00	0.00	0.00	0.18		0.00	0.00	0.00	0.00	0.00	0.00	0.00
NU	0.89	0.92	0.40	0.46	0.27	0.49	0.34	0.03	•	0.00	0.00	0.00	0.03		0.00	0.00	0.00	0.00	0.00	0.00	0.00
BC_C_	0.84	0.88	0.33	0.40	0.05	0.10	0.09	0.21	0.35	•	0.28	0.52	0.00		0.00	0.00	0.01	0.00	0.16	0.01	0.00
BC_H_	0.97	0.99	0.65	0.53	−0.01	<0.01	−0.01	0.25	0.47	0.02	•	0.18	0.01		0.01	0.16	0.11	0.62	0.80	0.32	0.00
AB	0.91	0.95	0.40	0.47	0.07	0.11	0.15	0.23	0.40	−0.01	0.05	•	0.00		0.00	0.01	0.05	0.00	0.12	0.00	0.00
SK	0.92	0.95	0.30	0.45	0.19	0.44	0.06	0.02	0.07	0.25	0.28	0.30	•		0.00	0.00	0.00	0.00	0.00	0.00	0.00
MB	0.79	0.86	0.21	0.36	0.33	0.42	0.19	0.28	0.37	0.34	0.27	0.31	0.19		•	0.00	0.01	0.00	0.00	0.00	0.00
ON	0.81	0.86	0.27	0.39	0.09	0.13	0.09	0.21	0.35	0.10	0.05	0.10	0.23		0.15	•	0.09	0.00	0.08	0.01	0.00
QC/NL_H_	0.91	0.95	0.27	0.39	0.19	0.29	0.22	0.31	0.47	0.12	0.19	0.09	0.32		0.18	0.06	•	0.00	0.04	0.01	0.01
MT	0.91	0.93	0.67	0.67	0.09	0.07	0.21	0.38	0.53	0.12	−0.05	0.15	0.50		0.54	0.19	0.40	•	0.71	0.12	0.00
WY	0.96	0.98	0.59	0.59	0.01	0.02	0.08	0.22	0.43	0.03	−0.09	0.05	0.30		0.29	0.06	0.20	−0.03	•	0.09	0.00
ID	0.99	1.00	0.92	0.71	0.12	0.07	0.52	0.41	0.60	0.11	0.12	0.17	0.54		0.41	0.14	0.36	0.06	0.12	•	0.00
CA_H_	0.98	1.00	0.84	0.66	0.67	0.82	0.80	0.69	0.76	0.65	0.87	0.70	0.69		0.37	0.55	0.49	0.85	0.84	0.98	•

SWE = Sweden; NOR = Norway; MNG = Mongolia; RUS = Russia; AK = Alaska; YT = Yukon; NT = Northwest Territories; NU = Nunavut; BC = British Columbia; AB = Alberta; SK = Saskatchewan; MB = Manitoba; ON = Ontario; QC/NL = Quebec-Labrador; MT = Montana; WY = Wyoming; ID = Idaho; CA = California; _C_ = Contemporary; _H_ = Historic.

SAMOVA identified negligible differences between φ_CT_ values as *K* increased for all analyses, with the exception of *K* = 2 for North American samples with CA_H_ and QC/NL_H_. This inflection point separated CA_H_ from the remaining samples. An inflection point of diminished values was also observed for among population within groups variation across all data combinations. The *K* value for which φ_ST_ values greatly decreased varied among data groups, however, a similar hierarchical pattern of genetic divisions was observed for all analyses (Table 3). The overall regional genetic groups delineated by φ_ST_ results were SWE-NOR, MNG, RUS, CA_H_, QC/NL_H_, MB, NT-NU-SK, and the remaining samples. Weaker relationships were found when populations were grouped according to geographic distribution in ANOVA.

**Table 3:** Results of delineated genetic groupings identified by SAMOVA for different population configurations.

Data Assemblage	Delineated Genetic Grouping	Variance Components	Percentage of Variance	φ-statistics	*P*
Eurasia, North America,CA_H_, & QC/NL_H_	*K* = 8 (SWE-NOR vs. MNG vs. RUS vs. CA_H_ vs.QC/NL_H_ vs. MB vs. NT-NU-SK vs. Rest)				
	Among groups	1.38	68.55	φ_CT_ = 0.68549	<0.001
	Among populations within groups	0.06	2.90	φ_SC_ = 0.09226	<0.001
	Within populations	0.57	28.55	φ_ST_ = 0.71451	<0.001
Eurasia & North America	*K* = 7 (SWE-NOR vs. MNG vs. RUS vs. MB vs.NT-NU vs. SK vs. Rest)				
	Among groups	1.39	68.88	φ_CT_ = 0.68876	<0.001
	Among populations within groups	0.06	2.95	φ_SC_ = 0.09490	<0.001
	Within populations	0.57	28.17	φ_ST_ = 0.71829	<0.001
North America, CA_H_ & QC/NL_H_	*K* = 5 (CA_H_ vs. QC/NL_H_ vs. MB vs. NT-NU-SK vs. Rest)				
	Among groups	0.43	35.54	φ_CT_ = 0.35540	<0.001
	Among populations within groups	0.07	5.49	φ_SC_ = 0.08510	<0.001
	Within populations	0.71	58.97	φ_ST_ = 0.41026	<0.001
North America	*K* = 3 (MB vs. NT-NU-SK vs. Rest)				
	Among groups	0.37	32.62	φ_CT_ = 0.32624	<0.001
	Among populations within groups	0.07	5.78	φ_SC_ = 0.08575	<0.001
	Within populations	0.70	61.60	φ_ST_ = 0.38401	0.001

SWE = Sweden; NOR = Norway; MNG = Mongolia; RUS = Russia; NT = Northwest Territories; NU = Nunavut; SK = Saskatchewan; MB = Manitoba; QC/NL_H_ = Historic Quebec-Labrador; CA_H_ = Historic California.

Mantel tests revealed a significant correlation between genetic and geographic matrices among sites (*r* = 0.190, *P* = 0.001), indicating that genetic differentiation was partially explained by isolation by distance. Similarly, isolation by distance was also found to be significant among North American localities (*r = *0.254, *P* = 0.033).

### Phylogenetic Analysis

**Figure 4:**
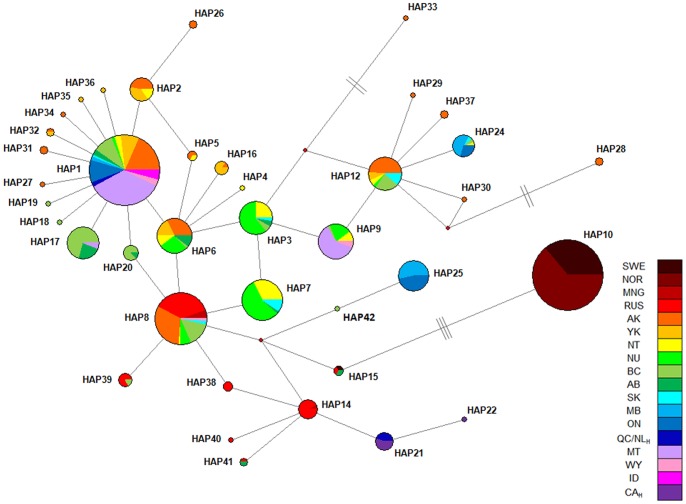
Median-joining network. Median-joining network of the mtDNA control region haplotypes for all samples combined. Haplotype size reflects relative frequency. Each branch represents one mutational step, unless otherwise noted. Black circles represent missing intermediate haplotypes.

The Bayesian phylogenetic tree (not shown) revealed low support (posterior probability <0.75) for all but one node in the derived wolverine phylogeny. The supported node (1.0 posterior probability) was found near the base of the tree, suggesting a single phylogenetic haplogroup across this carnivore’s Holarctic range. The predominance of low bootstrap values is likely the result of haplotypes being separated by single point mutations as revealed by the median-joining network (Figure 4). Although the mismatch distribution did not display the pattern of a typical Poisson distribution, it was unimodal (Figure S1), and consistent with a rapid range expansion. The variance (SSD = 0.0099; *P* = 0.636) and Harpending’s raggedness index (*r* = 0.026; *P* = 0.799) suggested that the observed mismatch distribution did not differ from the expected distribution of a population expansion model. Similarly, Fu’s *Fs* test showed a signal of population expansion across all wolverine samples (*Fs* = −10.688, *P* = 0.031). The median-joining network revealed a star shaped topology associated with Hap1. The main body of the network was characterized by several reticulations that included Hap3, Hap6, Hap7, Hap8, which primarily occurred in the northwestern North America and Russia (Figure 4). Additionally, the network identified eight mutational steps separating the two haplotypes predominately found in MB and ON, Hap24 and Hap25 (Figure 4).

### ABC Analysis

The selection of the most optimal model did not change whether or not a bottleneck was modeled within the MB-ON population. Results for both sets of analyses were comparable, thus we only present findings for the reference table with the population bottleneck for MB-ON. The DIYABC analysis revealed the third hypothesis of a single Beringia incursion during the LGM established the northwestern Pop1 and then followed by a west-to-east Holocene colonization was the most supported. The posterior probability of the logistic regression for scenario 3 was 1.0, with a type I error of 0.750 and a type II error of 0.147.

## Discussion

### Pleistocene Influence

Species with widespread contemporary distributions and/or highly mobile species are said to more likely to have occupied multiple glacial refugia during glaciations [98]. The star phylogeny and unimodal mismatch distribution, with non-significant sum of square deviations between observed and expected values, indicate a rapid range expansion of wolverines occurred from a single glacial refugium. The mismatch graph (Figure S1) did not display a standard Poisson distribution, but was similar to the mismatch distribution produced by Excoffier and Schneider [99] when an early population expansion also experienced a historic (400 generations ago) bottleneck. The further back in time bottlenecks take place, the longer the time period for increased genetic drift, resulting in increased variance of the mismatch distribution and higher frequencies of low difference classes (e.g. 0 and 1, [99]). This expansion event was also supported by a significantly negative Fu’s F*s* value for the whole data set. Although Fu’s F*s* statistic was not significant for individual regions, except for AK, the occurrence of negative values implies that there may be some deviation from neutrality for the northwestern region.

This expansion event from a single glacial refugium is in contrast to the phylogeographical structure of other cold adapted species such as woodland caribou (*Rangifer tarandus caribou*), a widely distributed and highly vagile ungulate. For woodland caribou a multimodal mismatch distribution pattern of mtDNA sequences [3] reflected postglacial expansions from three putative regions in North America [20]. Although data presented in this study were collected throughout the Holarctic range of wolverines, certain areas like the region between Scandinavia and the Russian Far East remained unsampled. In this study, Scandinavia represented a geographic outlier. Implementing a more systematic sampling scheme may identify new haplotypes or missing branching lineages, or change the frequency and distribution of known haplotypes. This may help resolve some of the reticulated haplotypes in the network and provide a more comprehensive assessment of the relationship between Hap10 (SWE-NOR) and adjacent haplotypes. Furthermore, severe population declines experienced by Scandinavian populations [26], with drift likely undermining our ability to resolve historic processes and links with other populations.

Our observation of low nucleotide diversity among sequences may be attributed to the amplification of a 318 bp fragment of the mtDNA control region. This sequence length is likely not sufficient to provide the necessary resolution needed for making phylogenetic inferences as observed with the unsupported phylogenetic tree. This small fragment size was used to allow us to expand our study and compare samples from existing databanks and get the largest distribution possible, and is clearly a compromise that does put some limits on our interpretations. The presence of low nucleotide diversity has also been observed in other mustelid species like pine marten (*Martes martes*, [13]) and fisher (*Martes pennanti*, [100]). In the case of pine marten, similarly truncated mtDNA control region sequences were also used (320 bp), with results suggesting European colonization from a single refugium following a recent glaciation [13]. Although a slightly longer sequence of the mtDNA control region was used for woodland caribou (429 bp, [20]), the finding of three highly supported phylogroups may be reflective of woodland caribou’s classification into different ecotypes. This shows that such a small mtDNA fragment can provide insight into phylogeographic processes.

If multiple refugial lineages persisted in present-day wolverine populations, we would expect to find clearly delineated subclades for each glacial refugium. Our network would also have numerous substitutions along branches connecting each of the subclades, as observed in the Nearctic clade d-loop network of the red fox [101]. The most differentiated haplotype was Hap10 found in SWE and NOR, but this haplotype was separated by only three mutations from Hap15 found in RU and AB. This step-wise mutation pattern does not provide evidence of longstanding genetic differences of animals isolated by multiple glacial refugia, but is suggestive of long range dispersal movements’ characteristic of wolveries. Single mutational step differences among control region haplotypes were also observed for the arctic fox (*Alopex lagopus*), with several haplotypes having a Holarctic distribution indicative of the long-distance dispersal capabilities of this carnivore [1]. Although our data suggests wolverines likely underwent a postglacial expansion from a single glacial refugium, the colonization pattern of this expansion and its effect on contemporary genetic structure of mtDNA remains unknown. A hypothesis testing approach could provide insights into different recolonization scenarios, and distinguish among these alternative hypotheses the most likely one.

### Hypothesis Testing of Population Divergence Scenarios

The most supported ABC model was a single incursion from Beringia during the LGM giving rise to a northwestern AK-YK-BC-AB-MT-WY-ID population, followed by a west-to-east stepping-stone divergence scenario during the Holocene. This pattern is suggestive of recolonization occurring in accordance with glacial retreat, where initial recolonization along the western coast of North America was followed by an inland recolonization pattern [102]. The inclusion of a recent bottleneck for the MB-ON populations did not change which scenario was best supported. Although scenario 3 had the highest posterior probability and a low type II error, summary statistics for all simulated data sets did not surround the observed data set, reflecting a poor fit by all four scenarios. This does not necessary mean that scenario 3 was erroneously selected, but that additional events may need to be incorporated in the proposed colonization patterns. One likely explanation for the poor fit may be the omission of a historic bottleneck across multiple populations as indicated by the mismatch distribution. While the mismatch analysis identified a historic bottleneck, additional information such as duration, time period, and geographic extent were not provided, but required for DIYABC simulations when modeling a bottleneck event. In addition, the DIYABC limitation that dispersal is absent among populations once they have diverged [103] may contribute to our poor fit between simulated and observed data sets. Both male and female wolverines have demonstrated extensive dispersal distances [51], [104], resulting in gene flow among diverged populations. Finally, our use of a short mtDNA fragment may not provide enough resolution to establish a good fit between simulated and observed data sets despite ABC suggesting some models are highly probable. We suggest that a cautionary approach be taken regarding ABC model choice, and this method be viewed as an exploratory tool [105].

### Historic and Contemporary Influences

The Bering Strait represents a barrier to wolverine movement across Eastern and Western Hemispheres. However, excluding the Eurasian samples from Mantel tests still produced a low *r* value (*r* = 0.293) for North America. Tomasik and Cook [55], and Cegelski et al. [49] found no support for IBD and suggested barriers may be influencing differentiation patterns. Extrinsic factors that appear to influence wolverine genetic structure include summer temperatures, spring snow cover and ecological changes associated with anthropogenic land use activities [36], [43], [52]–[54]. Additionally, our use of a maternal marker for a carnivore with male biased dispersal [50] would result in a stronger isolation by distance value particularly for the philopatric sex [106], but also be more apparent across all individuals. This absence of a stronger IBD further supports the influence of external factors on population differentiation.

Negligible differences between φ_CT_ statistics of SAMOVA for *K* values before and after inflection points were also observed by Schwartz et al. [60], while Frances [59] found an overall lack of geographic structure. Our SAMOVA analyses revealed lower *K* values being representative of SWE-NOR, CA_H_, MNG, QC/NL_H_, and MB. Schwartz et al. [60] also observed CA_H_ as a separate group for *K* >2, and that SWE-NOR separated from all other locations at *K* = 2. These lower *K* groupings represent peripheral regions of the wolverine’s contemporary distribution. Range peripheries are generally characterized by decreased density, ecologically marginal habitats and isolation [24], [107] that may bring about more frequent extinction-recolonization events, and likely result in a distinct diversity gradient as the species range edge is approached (see Peterman et al. [108]). Alternatively, more pronounced selection pressures in conjunction with reduced gene flow at range peripheries may lead to increased genetic distinctiveness of edge populations [23], [24].

Event though we observed spatial genetic structuring of mtDNA over small geographic scales, reflecting female philopatry [49], [55], we also found haplotypes displaying disjoined distributions. Hap15 was found in SWE and RUS, while Hap21 occurred in CA_H_ and QC/NL_H_ - the two most peripheral populations in North America. The presence of Hap21 and Hap15 in QC/NL_H_ and RUS samples in this study, and their independent observation in CA_H_ [60] and SWE [58] supports that our observation of these haplotypes are not the result of sequencing errors. The patchy distribution of Hap15 and Hap21 may reflect leptokurtic dispersal [109] following post-glacial colonization. Alternatively, both haplotypes may have been more widely distributed in the past but have since decreased in frequency or become lost from adjacent regions due to direct human persecution in both hemispheres [26], [65], extensive range contractions in North America [52], and random effects of genetic drift. To distinguish between long-distance dispersal and fragmentation, additional sampling is needed between Scandinavia and the Russian Far East, along with the inclusion of supplementary history samples from adjacent regions of CA_H_ and QC/NL_H_.

Manitoba and Ontario were characterized by Hap1, Hap24, and Hap25, with the latter two haplotypes occurring almost exclusively in these two regions (Figure 3). The separation of MB and ON by both SAMOVA and pairwise Φ_ST_ estimates was unexpected based on the very different composition of MB and ON in comparison to the other regions, and that microsatellite data group MB and ON as a separate genetic cluster from the panmictic northwest population [51]. Based on this, MB and ON should be pooled together as a separate genetic group. Contrasting haplotype frequencies among the three regions likely explains why MB and ON were separated into different genetic clusters [51]. In particular, the higher frequency of Hap1 in ON (65%) compared to MB (19%) is presumed to have influenced the grouping of ON with other regions also having high Hap1 frequencies. This high Hap1 frequency in ON may have occurred as a result of samples consisting of closely related individuals than expected by chance. The majority of samples from ON were collected using baited hair snares encompassing 2 000 km^2^ [110], increasing the likelihood of close relatives. Alternatively, substantial population declines during the early 1900s [65], where densities have historically been considered low, likely increased the effects of genetic drift and strong female philopatry continuing to maintain altered haplotype frequencies [45], [55], [60].

### Conservation Implications

Pairwise Φ_ST_ estimates pooled QC/NL_H_ with contemporary ON. This grouping is likely due to Hap21 (QC/NL_H_) and Hap25 (ON) being derived from the same missing intermediate haplotype (Figure 4), and the high frequency of Hap1 in both regions. This genetic clustering of QC/NL_H_ with the extant peripheral cluster of MB-ON brings into question the classification of wolverines from Quebec-Labrador as the ‘eastern’ population. This potential grouping will also influence the selection of a source population for the proposed translocation of wild animals as outlined in the national recovery plan for the eastern wolverine population in Quebec-Labrador [38]. However, this grouping does not preclude the possibility of QC/NL_H_ with MB-ON being considered a designable unit. For a population to be identified as a designatable unit (DU), the population needs to be recognized as being discrete and evolutionary significant [111]. Using both mtDNA and microsatellites, Zigouris et al. [51] found MB-ON formed a separate genetic cluster from core regions (NT-NU-SK). In addition, genetic analysis of multilocus genes found in the major histocompatibility complex (MHC) for wolverines across their Canadian range revealed a duplicated DRB exon 2 with the peptide binding region having a significant excess of non-synonymous substitutions - indicative of positive selection acting on MHC of wolverines [112]. Further research is needed on wolverines along their eastern range edge in North America, including combining ecological data with genetic markers under natural selection [113], [114], in order to appropriately define a population as a DU [111].

## Conclusions

In contrast to previous glacial refugia studies of arctic species (e.g. [18]–[20]), we found no molecular evidence of wolverines inhabiting multiple glacial refugia during the last glacial maximum. In addition, approximate Bayesian computations supported a wolverine colonization of North American where individuals followed retreating glaciers. Even with molecular evidence of a single expansion event, significant subdivisions of population genetic structure over small spatial scales were observed. This genetic structure reflected historic population declines throughout the wolverine’s Holarctic range, as indicated by the mismatch distribution, along with the influence of subsequent genetic drift and strong female philopatry (e.g. [45], [55], [60]). We acknowledge that the mtDNA analyses in this study were based on a short sequence fragment of the control region, restricting the resolution needed for us to make conclusive inferences. However, these data provide some insights into the post-glacial colonization and phylogeographic relationships among contemporary wolverine populations. In particular, our observation of low genetic differentiation between QC/NL_H_ and ON, which puts into question the designation of Quebec-Labrador as the ‘eastern’ population [115]. These underlining genetic associations among regions highlight key areas and questions where future research should to focus on, including using longer mtDNA fragment or alternative markers like single nucleotide polymorphisms (SNPs) to obtain deeper resolution of phylogeographic history. We also suggest further research be undertaken with functional markers to investigate the possibility of local adaptation among the different genetic clusters. This information will have strong implications for the identification of designatable units [111], and future conservation and recovery activities of wolverines, particularly at the eastern periphery of their North American range.

## Supporting Information

Figure S1 **Mismatch distribution.** Pairwise mismatch distribution performed on mtDNA haplotypes among individuals. Bars represent observed values, a solid line represents the expected distribution according to the sudden expansion model, and dotted lines show ±95% confidence intervals.
(TIF)

Table S1 **Catalogue information of all wolverine samples obtained from collections and if samples were sequenced.**
(DOC)

Table S2 **Minimum and maximum limits and associated coditions of parameters used in DIYABC analyses.**
(DOC)

Table S3 **Control region haplotype labels discussed in this study and their corresponding designation(s) as identified in previous studies. Sequential numbering of each haplotype is reflective of when it was first published in the literature.**
(DOC)

Table S4 **Variable nucleotide positions of identified haplotypes are denoted according to their location within the 318 bp portion of the D-loop mtDNA control region.**
(DOC)

Table S5 **Percent frequencies of wolverine mtDNA control region haplotypes within and across regions.**
(DOC)
