# Supplementary figures and images for: Phylogeography and Post-Glacial Recolonization in Wolverines (Gulo gulo) from across Their Circumpolar Distribution
Source: PLoS One. 2013 Dec 30;8(12):e83837. doi: 10.1371/journal.pone.0083837 (PMC3875487; doi:10.1371/journal.pone.0083837)

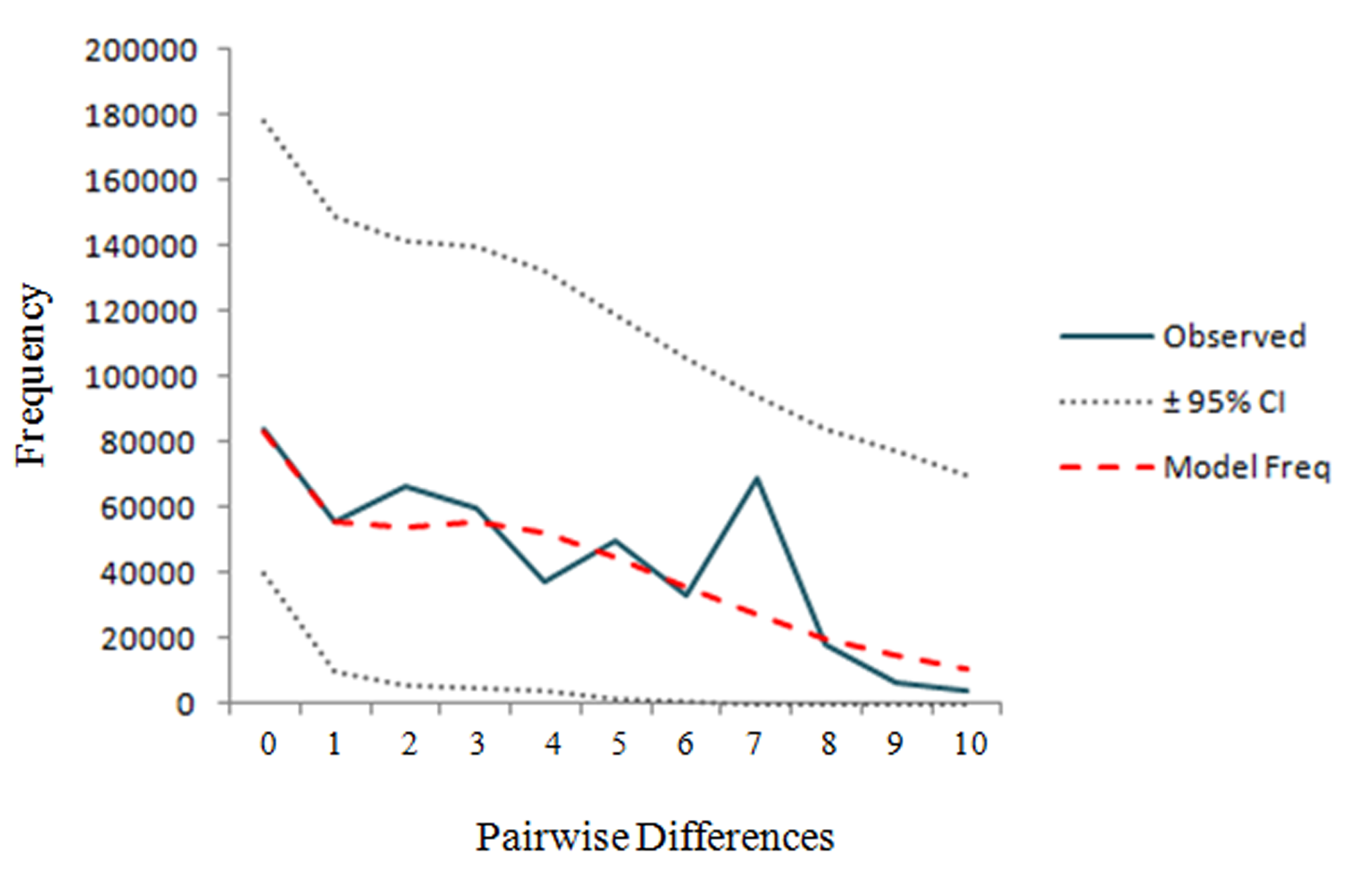

Supplement: Figure S1 — Mismatch distribution. Pairwise mismatch distribution performed on mtDNA haplotypes among individuals. Bars represent observed values, a solid line represents the expected distribution according to the sudden expansion model, and dotted lines show ±95% confidence intervals. (TIF) [file pone.0083837.s001.tif]
