# Supplementary material for: Phylogeography and Post-Glacial Recolonization in Wolverines (Gulo gulo) from across Their Circumpolar Distribution
Source: PLoS One. 2013 Dec 30;8(12):e83837. doi: 10.1371/journal.pone.0083837 (PMC3875487; doi:10.1371/journal.pone.0083837)
Supplement: Table S1 — Catalogue information of all wolverine samples obtained from collections and if samples were sequenced. (DOC) [file pone.0083837.s002.doc]

| **Provider** | **Catalogue Number** | **Collection Date** | **Locality** | **Province** | **Sequenced** |
| --- | --- | --- | --- | --- | --- |
| University of Colorado Museum of Natural History | 10678 | 01 January 1910 | Vancouver Island | British Columbia | Yes |
| Museum of Comparative Zoology, Harvard University | 18935 | 01 August 1920 | Athabaska Lake | Saskatchewan | Yes |
| Museum of Comparative Zoology, Harvard University | BANGS-7429 | Summer 1897 | Okak | Labrador | Yes |
| Museum of Comparative Zoology, Harvard University | BANGS-7430 | Summer 1897 | Okak | Labrador | Yes |
| Museum of Comparative Zoology, Harvard University | BOM-7483 | 1898 | Big Bay | Labrador | Yes |
| Museum of Comparative Zoology, Harvard University | BOM-7484 | 30 January 1899 | Kippokak Bay | Labrador | Yes |
| Museum of Comparative Zoology, Harvard University | BANGS-8881 | 24 October 1898 | Black Bay | Labrador | Yes |
| Museum of Comparative Zoology, Harvard University | BANGS-8882 | 9 January 1899 | Black Bay | Labrador | Yes |
| Museum of Comparative Zoology, Harvard University | BANGS-8962 | 10 November 1899 | Lance au Loup | Labrador | Yes |
| National Museum of Natural History | USNM A23142 | Pre 1900 | Ungava | Quebec | Yes |
| National Museum of Natural History | USNM 188245 | 24 April 1888 | Godbout | Quebec | No |
| National Museum of Natural History | USNM 189000 | 1883 | Near Godbout | Quebec | Yes |
| National Museum of Natural History | USNM A23256 | Pre 1900 | Ungava | Quebec | Yes |
| National Museum of Natural History | USNM 14781 | Pre 1900 | Ungava, Rapids | Quebec | Yes |
| National Museum of Natural History | USNM A23254 | 21 February 1889 | Ungava | Quebec | No |
| National Museum of Natural History | USNM 188244 | 1889 | Godbout | Quebec | Yes |
| National Museum of Natural History | USNM 141911 | Pre 1900 | Ungava | Quebec | Yes |
| The Museum of Vertebrate Zoology, Berkeley | 33270 | 1916 | Lower Iskut River near junction with Stikine River | British Columbia | No |
| The Museum of Vertebrate Zoology, Berkeley | 33271 | 1916 | Lower Iskut River near junction with Stikine River | British Columbia | Yes |
| The Museum of Vertebrate Zoology, Berkeley | 43625 | 11 April 1929 | Indianpoint Lake, Cariboo District | British Columbia | No |
| The Museum of Vertebrate Zoology, Berkeley | 33269 | 1916 | Lower Iskut River near junction with Stikine River | British Columbia | Yes |
| The Museum of Vertebrate Zoology, Berkeley | 43631 | Winter 1926-1927 | McClary Lake region | British Columbia | No |
| The Museum of Vertebrate Zoology, Berkeley | 43634 | Winter 1927-1928 | McClary Lake region | British Columbia | Yes |
| The Museum of Vertebrate Zoology, Berkeley | 34396 | Winter 1923-1924 | 97 km W Carcross | Yukon | No |
| The Museum of Vertebrate Zoology, Berkeley | 159320* | Winter 1974-1975 | vicinity of Paint Lake, 30 km S Thompson | Manitoba | Yes |
| The Museum of Vertebrate Zoology, Berkeley | 43632 | Spring 1926-1927 | McClary Lake region | British Columbia | No |
| The Museum of Vertebrate Zoology, Berkeley | 43633 | Winter 1926-1927 | McClary Lake region | British Columbia | Yes |
| The Museum of Vertebrate Zoology, Berkeley | 33267 | Winter 1916-1917 | Lower Iskut River near junction with Stikine River | British Columbia | Yes |
| The Museum of Vertebrate Zoology, Berkeley | 33268 | Winter 1916-1917 | Lower Iskut River near junction with Stikine River | British Columbia | Yes |
| Royal Ontario Museum | 35.4.3.1 | 1920 | Thunder Bay | Ontario | No |
| Royal Ontario Museum | 16261 | 1944 | Qamani'tuaq (Baker Lake), Keewatin | Nunavut | No |
| Royal Ontario Museum | 31.2.16.9 | January 1930 | White River | Yukon | Yes |
| Royal Ontario Museum | 32.10.16.21 | 26 December 1931 | Yukon River | Yukon | No |
| Royal Ontario Museum | 32.10.16.22 | 5 March 1932 | White River | Yukon | Yes |
| Royal Ontario Museum | 32.10.16.23 | 10 February 1931 | White River | Yukon | Yes |
| Royal Ontario Museum | 32.10.16.24 | 1 March 1932 | White River | Yukon | Yes |
| Royal Ontario Museum | 32.10.16.25 | 1 December 1931 | White River | Yukon | Yes |
| Royal Ontario Museum | 32.10.16.27 | March 1931 | White River | Yukon | No |
| Royal Ontario Museum | 32.10.16.28 | March 1931 | White River | Yukon | No |

*Specimen was grouped with contemporary samples as it was collected post-1945.
