# Supplementary material for: Phylogeography and Post-Glacial Recolonization in Wolverines (Gulo gulo) from across Their Circumpolar Distribution
Source: PLoS One. 2013 Dec 30;8(12):e83837. doi: 10.1371/journal.pone.0083837 (PMC3875487; doi:10.1371/journal.pone.0083837)
Supplement: Table S2 — Minimum and maximum limits and associated coditions of parameters used in DIYABC analyses. (DOC) [file pone.0083837.s003.doc]

| **Parameter** | **Parameter Symbol** | **Minimum** | **Maximum** | **Conditions** | **References** |
| --- | --- | --- | --- | --- | --- |
| Ancestral population (Beringia) | NA | 1000 | 10000 |  |  |
| *Ne* sample 1 (Pop1) - AK, YK, BC, AB, MT, WY, ID | N1 | 1000 | 10000 |  |  |
| *Ne* sample 2 (Pop2) - NU, NT, SK | N2 | 1000 | 10000 |  | Slough [65] |
| *Ne* sample 3 (Pop3) - MB, ON | N3 | 50 | 1000 |  | Slough [65] |
| *Ne* sample 4 (Pop4) - QC/NL | N4 | 50 | 75 |  | Slough [65] |
| *Ne* sample 5 (Pop 5) - RUS, MNG | N5 | 1000 | 10000 |  | Slough [65] |
| *Ne* sample 3 prior to bottleneck - not sampled | N6 | 50 | 1000 | N6 > N3 | Slough [65] |
| *Ne* of ancestral North American Population | N7 | 1000 | 10000 | N7 > N1; N7 > N2; N7 > N4; N7 > N6 |  |
| *Ne*  sample 3 during the bottleneck | NF3 | 6 | 125 | NF1 < N3 |  |
| Start of bottleneck (generations) | tc | 22.3 | 22.3 |  | Slough [65] |
| Duration of bottleneck (generations) | db | 12.5 | 12.5 |  | Slough [65] |
| Divergence of Pop4 for Hypothesis 3 (generations) | td | 28 | 28 |  |  |
| Divergence of populations since the start of the Holocene and European colonization (generations) | t1, t2, t3 | 125 | 2900 | t1 < t2; t2 < t3 | Dykoski et al. [1], Cole [2] |
| Divergence from ancestral population during LGM - crossing of Beringia (generations) | ta, ta1, ta2 | 3125 | 15000 | ta1 < ta2 | Elias et al. [3], Pitulko et al. [4] |

1. Dykoski CA, Edwards RL, Cheng H, Yuan D, Cai Y et al. (2005) A high-resoltion, absolute-dated Holocene and deglacial Asian monsoon record from Dongge Cave, China. Earth Planet Sc Lett 233: 71-86.

2. Cole L (2010) Vegetation response to early Holocene warming as an analog for current and future changes. Conserv Biol 24: 29-37.

3. Elias SA, Short SK, Nelson CH, Birks HH (1996) Life and times of the Bering land bridge. Nature 382: 60-63.

4. Pitulko VV, Nikolsky PA, Girya EY, Basilyan AE, Tumskoy VEW et al. (2004) The Yana RHS site: humans in the arctic before the Last Glacial Maximum. Science 303: 52-56.
