# Supplementary material for: Phylogeography and Post-Glacial Recolonization in Wolverines (Gulo gulo) from across Their Circumpolar Distribution
Source: PLoS One. 2013 Dec 30;8(12):e83837. doi: 10.1371/journal.pone.0083837 (PMC3875487; doi:10.1371/journal.pone.0083837)
Supplement: Table S3 — Control region haplotype labels discussed in this study and their corresponding designation(s) as identified in previous studies. Sequential numbering of each haplotype is reflective of when it was first published in the literature. (DOC) [file pone.0083837.s004.doc]

| This Study | Wilson et al. 2000 | Walker et al. 2001 | Chappell et al. 2004 | Tomasik and Cook 2005 | Cegelski et al. 2006 | Schwartz et al. 2007 | Frances 2008 | Accession Numbers |
| --- | --- | --- | --- | --- | --- | --- | --- | --- |
| Hap1 | A |  | A | B | A |  | Hap1 | AF210090 |
| Hap2 | B |  | B | I |  |  | Hap12 | AF210094 |
| Hap3 | C |  | C | G | C |  | Hap10 | AF210107 |
| Hap4 | D |  | D | P |  |  | Hap27 | AF210097 |
| Hap5 | E |  | E | K |  |  | Hap26 | AF210098 |
| Hap6 | F |  | F | F | F |  | Hap6 | AF210105 |
| Hap7 | G |  | G | E |  |  | Hap9 | AF210106 |
| Hap8 | H |  | H | A | H |  | Hap2 | AF210130 |
| Hap9 | I |  | I | H |  |  | Hap11 | AF210112 |
| Hap10 |  | Scandinavia |  |  |  |  | Hap35 | AF245496 |
| Hap11* |  |  |  | C |  |  |  | AF56914 |
| Hap12 |  |  |  | D |  |  | Hap3 | AF55403 |
| Hap13* |  |  |  | J |  |  |  | AF56912 |
| Hap14 |  |  |  | L |  |  | Hap31 | AF52358 |
| Hap15 |  |  |  | M |  |  | Hap32 | AF52388 |
| Hap16 |  |  |  | N |  |  | Hap13 | AF15901 |
| Hap17 |  |  |  |  | L |  | Hap8 | see Cegelski et al. [49] |
| Hap18 |  |  |  |  | M |  | Hap28 | " |
| Hap19 |  |  |  |  | N |  | Hap29 | " |
| Hap20 |  |  |  |  | O |  | Hap30 | " |
| Hap21 |  |  |  |  |  | Cali1 | Hap33 | AY880313 |
| Hap22 |  |  |  |  |  | Cali2 | Hap34 | AY880314 |
| Hap23* |  |  |  |  |  | Mng1 |  | AY880315 |
| Hap24 |  |  | K |  |  |  | Hap23 | AY185168 |
| Hap25 |  |  | J |  |  |  | Hap22 | AY185167 |
| Hap26 |  |  |  |  |  |  | Hap4 | EU812347 |
| Hap27 |  |  |  |  |  |  | Hap5 | EU812353 |
| Hap28 |  |  |  |  |  |  | Hap7 | EU812365 |
| Hap29 |  |  |  |  |  |  | Hap14 | EU812407 |
| Hap30 |  |  |  |  |  |  | Hap15 | EU812416 |
| Hap31 |  |  |  |  |  |  | Hap16 | EU812422 |
| Hap32 |  |  |  |  |  |  | Hap17 | EU812424 |
| Hap33 |  |  |  |  |  |  | Hap18 | EU812430 |
| Hap34 |  |  |  |  |  |  | Hap19 | EU812431 |
| Hap35 |  |  |  |  |  |  | Hap21 | EU812442 |
| Hap36 |  |  |  |  |  |  | Hap24 | EU812451 |
| Hap37 |  |  |  |  |  |  |  | EU812432 |
| Hap38 |  |  |  |  |  |  |  | KC182788 |
| Hap39 |  |  |  |  |  |  |  | KC182789 |
| Hap40 |  |  |  |  |  |  |  | KC182790 |
| Hap41 |  |  |  |  |  |  |  | KC182791 |
| Hap42 |  |  |  |  |  |  |  | KC182792 |

Amalgamated haplotypes (Hap11 → Hap1; Hap13 → Hap6; Hap23 → 14) when 360 bp control region fragment was reduced to 318 bp to include sequence data from Walker et al. [15].
