# Supplementary material for: Phylogeography and Post-Glacial Recolonization in Wolverines (Gulo gulo) from across Their Circumpolar Distribution
Source: PLoS One. 2013 Dec 30;8(12):e83837. doi: 10.1371/journal.pone.0083837 (PMC3875487; doi:10.1371/journal.pone.0083837)
Supplement: Table S4 — Variable nucleotide positions of identified haplotypes are denoted according to their location within the 318 bp portion of the D-loop mtDNA control region. (DOC) [file pone.0083837.s005.doc]

|  | **Variable Nucleotide Position** | | | | | | | | | | | | | | | | | | | | | | | | | | | |
| --- | --- | --- | --- | --- | --- | --- | --- | --- | --- | --- | --- | --- | --- | --- | --- | --- | --- | --- | --- | --- | --- | --- | --- | --- | --- | --- | --- | --- |
|  |  |  |  |  |  |  |  |  |  |  |  |  | 1 | 1 | 1 | 1 | 1 | 1 | 1 | 1 | 1 | 2 | 2 | 2 | 2 | 2 | 2 | 2 |
|  |  | 1 | 1 | 2 | 2 | 4 | 5 | 5 | 5 | 6 | 6 | 8 | 1 | 2 | 2 | 2 | 4 | 5 | 7 | 8 | 8 | 0 | 0 | 0 | 0 | 1 | 8 | 9 |
| **Haplotype** | 8 | 0 | 8 | 6 | 9 | 7 | 6 | 7 | 9 | 0 | 5 | 3 | 1 | 3 | 4 | 5 | 7 | 3 | 8 | 5 | 7 | 0 | 1 | 6 | 9 | 5 | 8 | 3 |
| Hap1 | **G** | **A** | **C** | **C** | **A** | **T** | **A** | **T** | **C** | **C** | **─** | **A** | **T** | **─** | **─** | **T** | **C** | **C** | **A** | **G** | **T** | **T** | **C** | **T** | **G** | **G** | **A** | **T** |
| Hap2 | **•** | • | • | • | • | • | • | **C** | • | • | • | • | • | • | • | • | • | • | • | • | • | • | • | • | • | • | • | • |
| Hap3 | **•** | • | • | • | • | • | • | • | • | **T** | • | • | • | • | • | • | **T** | • | • | • | • | • | • | • | • | • | • | • |
| Hap4 | **•** | • | **A** | • | • | • | • | • | • | • | • | • | • | • | • | • | **T** | • | • | • | • | • | • | • | • | • | • | • |
| Hap5 | **•** | • | • | • | • | • | • | **C** | • | • | • | • | • | • | • | • | **T** | • | • | • | • | • | • | • | • | • | • | • |
| Hap6 | **•** | • | • | • | • | • | • | • | • | • | • | • | • | • | • | • | **T** | • | • | • | • | • | • | • | • | • | • | • |
| Hap7 | **•** | • | • | • | • | • | • | • | • | **T** | • | • | • | • | • | • | **T** | • | • | **A** | • | • | • | • | • | • | • | • |
| Hap8 | **•** | • | • | • | • | • | • | • | • | • | • | • | • | • | • | • | **T** | • | • | **A** | • | • | • | • | • | • | • | • |
| Hap9 | **•** | • | • | • | • | • | • | • | • | **T** | • | • | • | **C** | • | • | **T** | • | • | • | • | • | • | • | • | • | • | • |
| Hap10 | **A** | **T** | • | • | • | • | **─** | • | • | • | • | • | • | • | • | • | **T** | • | • | **A** | • | **C** | • | • | • | • | **G** | • |
| Hap12 | • | • | • | • | • | • | • | • | • | **T** | • | • | • | **C** | • | • | **T** | • | **G** | • | • | • | • | • | • | • | • | • |
| Hap14 | • | • | • | • | • | • | • | • | • | • | • | • | • | • | • | • | **T** | • | • | **A** | • | **C** | **T** | • | • | • | • | • |
| Hap15 | • | • | • | • | • | • | • | • | • | • | • | • | • | • | • | • | **T** | • | • | **A** | • | **C** | • | • | • | • | **G** | • |
| Hap16 | • | • | • | • | • | • | • | • | • | • | • | • | **C** | • | • | • | **T** | • | • | • | • | • | • | • | • | • | • | • |
| Hap17 | • | • | • | • | • | • | • | • | • | • | • | • | • | • | • | • | • | • | • | • | • | • | **T** | • | • | • | • | • |
| Hap18 | • | • | • | • | • | • | • | • | • | • | • | • | • | • | • | • | • | • | **G** | • | • | • | • | • | • | • | • | • |
| Hap19 | • | • | • | • | • | • | • | • | • | • | • | **G** | • | • | • | • | • | • | • | • | • | • | • | • | • | • | • | • |
| Hap20 | • | • | • | • | • | • | • | • | • | • | • | • | • | • | • | • | • | • | • | **A** | • | • | • | • | • | • | • | • |
| Hap21 | • | • | • | • | • | **C** | • | • | • | • | • | • | • | • | • | • | **T** | • | • | **A** | • | **C** | **T** | • | • | • | • | • |
| Hap22 | • | • | • | • | • | **C** | • | • | • | • | • | • | • | • | • | • | **T** | **T** | • | **A** | • | **C** | **T** | • | • | • | • | • |
| Hap24 | • | • | • | • | • | • | • | • | • | **T** | • | • | • | **C** | **C** | • | **T** | • | **G** | • | • | • | • | • | • | • | • | • |
| Hap25 | • | • | • | • | • | **C** | • | • | • | • | • | • | • | • | • | • | **T** | • | • | **A** | • | **C** | • | **C** | • | • | • | • |
| Hap26 | • | • | • | • | • | • | • | **C** | • | • | • | • | • | **C** | • | • | • | • | • | • | • | • | • | • | • | • | • | • |
| Hap27 | • | • | **G** | • | • | • | • | • | • | • | • | • | • | • | • | • | • | • | • | • | • | • | • | • | • | • | • | • |
| Hap28 | • | • | **G** | **T** | • | • | • | • | • | • | • | • | • | **C** | • | • | **T** | • | **G** | • | • | • | • | • | • | • | • | • |
| Hap29 | • | • | • | • | • | • | • | • | • | **T** | • | • | • | **C** | • | • | **T** | • | **G** | • | • | • | • | • | • | • | • | **G** |
| Hap30 | • | • | **─** | • | • | • | • | • | • | **T** | • | • | • | **C** | • | • | **T** | • | **G** | • | • | • | • | • | • | • | • | • |
| Hap31 | • | • | • | • | • | • | • | • | • | • | • | • | • | • | • | • | • | • | • | • | • | • | • | • | • | **A** | • | • |
| Hap32 | • | • | • | • | • | • | • | • | • | • | **A** | • | • | • | • | • | • | • | • | • | • | • | • | • | • | • | • | • |
| Hap33 | • | • | • | • | • | • | • | • | • | **T** | • | • | • | • | • | • | **T** | • | **G** | • | • | • | • | • | **A** | **A** | • | • |
| Hap34 | • | • | • | • | • | • | • | • | **T** | • | • | • | • | • | • | • | • | • | • | • | • | • | • | • | • | • | • | • |
| Hap35 | • | • | • | **T** | • | • | • | • | • | • | • | • | • | • | • | • | • | • | • | • | • | • | • T | • | • | • | • | • |
| Hap36 | • | • | • | • | **─** | • | • | • | • | • | • | • | • | • | • | • | • | • | • | • | • | • | • | • | • | • | • | • |
| Hap37 | • | • | • | • | • | • | • | • | • | **T** | • | • | • | **C** | • | **─** | **T** | • | **G** | • | • | • | • | • | • | • | • | • |
| Hap38 | • | • | • | • | • | • | • | • | • | • | • | • | • | • | • | • | **T** | • | • | **A** | • | • | **T** | • | • | • | • | • |
| Hap39 | • | • | • | • | • | • | • | • | • | • | • | • | • | • | • | • | **T** | • | • | **A** | **C** | • | • | • | • | • | • | • |
| Hap40 | **A** | • | • | • | • | • | • | • | • | • | • | • | • | • | • | • | **T** | • | • | **A** | • | **C** | **T** | • | • | • | • | • |
| Hap41 | • | • | • | • | • | • | • | • | • | • | • | • | • | **C** | • | • | **T** | • | • | **A** | • | **C** | **T** | • | • | • | • | • |
| Hap42 | • | • | • | • | • | • | • | • | • | • | • | • | • | • | • | • | **T** | • | • | **A** | • | **C** | • | **C** | • | • | • | • |
