# Supplementary material for: Phylogeography and Post-Glacial Recolonization in Wolverines (Gulo gulo) from across Their Circumpolar Distribution
Source: PLoS One. 2013 Dec 30;8(12):e83837. doi: 10.1371/journal.pone.0083837 (PMC3875487; doi:10.1371/journal.pone.0083837)
Supplement: Table S5 — Percent frequencies of wolverine mtDNA control region haplotypes within and across regions. (DOC) [file pone.0083837.s006.doc]

|  | **Within Region Frequency (%)** | | | | | | | | | | | | | | | | | | | |  |
| --- | --- | --- | --- | --- | --- | --- | --- | --- | --- | --- | --- | --- | --- | --- | --- | --- | --- | --- | --- | --- | --- |
| **Haplotype** | SWE | NOR | MNG | RUS | AK | YT_C_ | YT_H_ | NT | NU | BC_C_ | BC­_H_ | AB | SK_C_ | MB | ON | QC/NL­_H_ | MT | WY | ID | CA­_H_ | **Total Frequency (%) Across Regions** |
| Hap1 |  |  |  |  | 43 | 53 | 60 | 19 | 6 | 31 | 86 | 31 | 19 | 19 | 65 | 62 | 83 | 77 | 100 |  | 36 |
| Hap2 |  |  |  |  | 6 | 14 |  | 6 |  |  |  |  |  |  |  |  |  |  |  |  | 2 |
| Hap3 |  |  |  |  |  |  |  | 17 | 26 | 3 |  | 7 | 6 |  |  |  |  |  |  |  | 4 |
| Hap4 |  |  |  |  |  |  |  | 2 |  |  |  |  |  |  |  |  |  |  |  |  | <1 |
| Hap5 |  |  |  |  | 1 |  |  | 2 |  |  |  |  |  |  |  |  |  |  |  |  | <1 |
| Hap6 |  |  |  |  | 9 | 14 |  | 7 | 14 | 1 |  | 15 |  |  |  |  |  |  |  |  | 4 |
| Hap7 |  |  |  |  |  |  |  | 32 | 36 |  |  | 4 | 31 |  |  |  |  |  |  |  | 5 |
| Hap8 |  |  | 83 | 59 | 18 |  |  | 2 | 7 | 15 |  |  | 13 |  |  |  |  | 8 |  |  | 9 |
| Hap9 |  |  |  |  |  |  |  | 7 | 10 | 1 |  |  |  |  |  |  | 16 | 15 |  |  | 4 |
| Hap10 | 98 | 100 |  |  |  |  |  |  |  |  |  |  |  |  |  |  |  |  |  |  | 17 |
| Hap12 |  |  |  |  | 12 | 2 | 40 | 4 | 1 | 8 | 14 |  | 25 |  |  |  |  |  |  |  | 4 |
| Hap14 |  |  | 17 | 20 |  |  |  |  |  |  |  |  |  |  |  |  |  |  |  |  | 1 |
| Hap15 | 2 |  |  | 2 |  |  |  |  |  |  |  | 4 |  |  |  |  |  |  |  |  | <1 |
| Hap16 |  |  |  |  | 1 | 11 |  |  |  |  |  |  |  |  |  |  |  |  |  |  | 1 |
| Hap17 |  |  |  |  |  |  |  |  |  | 28 |  | 31 |  |  |  |  | 1 |  |  |  | 3 |
| Hap18 |  |  |  |  |  |  |  |  |  | 1 |  |  |  |  |  |  |  |  |  |  | <1 |
| Hap19 |  |  |  |  |  |  |  |  |  | 1 |  |  |  |  |  |  |  |  |  |  | <1 |
| Hap20 |  |  |  |  |  |  |  |  |  | 8 |  | 4 |  |  |  |  |  |  |  |  | 1 |
| Hap21 |  |  |  |  |  |  |  |  |  |  |  |  |  |  |  | 38 |  |  |  | 86 | 1 |
| Hap22 |  |  |  |  |  |  |  |  |  |  |  |  |  |  |  |  |  |  |  | 14 | <1 |
| Hap24 |  |  |  |  |  |  |  | 2 |  | 1 |  |  | 6 | 29 | 9 |  |  |  |  |  | 2 |
| Hap25 |  |  |  |  |  |  |  |  |  |  |  |  |  | 52 | 26 |  |  |  |  |  | 3 |
| Hap26 |  |  |  |  | 1 |  |  |  |  |  |  |  |  |  |  |  |  |  |  |  | <1 |
| Hap27 |  |  |  |  | 1 |  |  |  |  |  |  |  |  |  |  |  |  |  |  |  | <1 |
| Hap28 |  |  |  |  | 1 |  |  |  |  |  |  |  |  |  |  |  |  |  |  |  | <1 |
| Hap29 |  |  |  |  | 1 |  |  |  |  |  |  |  |  |  |  |  |  |  |  |  | <1 |
| Hap30 |  |  |  |  | 1 |  |  |  |  |  |  |  |  |  |  |  |  |  |  |  | <1 |
| Hap31 |  |  |  |  | 1 |  |  |  |  |  |  |  |  |  |  |  |  |  |  |  | <1 |
| Hap32 |  |  |  |  | 1 | 2 |  |  |  |  |  |  |  |  |  |  |  |  |  |  | <1 |
| Hap33 |  |  |  |  | 1 |  |  |  |  |  |  |  |  |  |  |  |  |  |  |  | <1 |
| Hap34 |  |  |  |  | 1 |  |  |  |  |  |  |  |  |  |  |  |  |  |  |  | <1 |
| Hap35 |  |  |  |  |  | 2 |  |  |  |  |  |  |  |  |  |  |  |  |  |  | <1 |
| Hap36 |  |  |  |  |  | 2 |  |  |  |  |  |  |  |  |  |  |  |  |  |  | <1 |
| Hap37 |  |  |  |  | 1 |  |  |  |  |  |  |  |  |  |  |  |  |  |  |  | <1 |
| Hap38 |  |  |  | 6 |  |  |  |  |  |  |  |  |  |  |  |  |  |  |  |  | <1 |
| Hap39 |  |  |  | 9 |  |  |  |  |  | 1 |  |  |  |  |  |  |  |  |  |  | 1 |
| Hap40 |  |  |  | 2 |  |  |  |  |  |  |  |  |  |  |  |  |  |  |  |  | <1 |
| Hap41 |  |  |  | 2 |  |  |  |  |  |  |  | 4 |  |  |  |  |  |  |  |  | <1 |
| Hap42 |  |  |  |  |  |  |  |  |  | 1 |  |  |  |  |  |  |  |  |  |  | <1 |
| *n* | *62* | *108* | *6* | *54* | *148* | *49* | *5* | *53* | *81* | *86* | *7* | *26* | *16* | *31* | *54* | *13* | *148* | *13* | *15* | *7* |  |

SWE = Sweden; NOR = Norway; MNG = Mongolia; RUS = Russia; AK = Alaska; YT = Yukon; NT = Northwest Territories; NU = Nunavut; BC = British Columbia; AB = Alberta; SK = Saskatchewan; MB = Manitoba; ON = Ontario; QC/NL = Quebec-Labrador; MT = Montana; WY = Wyoming; ID = Idaho; CA = California; _C_ = Contemporary; _H_ = Historic; *n =* sample size
